# Supplementary material for: Rapid turnover of life-cycle-related genes in the brown algae
Source: Genome Biol. 2019 Feb 14;20:35. doi: 10.1186/s13059-019-1630-6 (PMC6374913; doi:10.1186/s13059-019-1630-6)
Supplement: Supplementary file 3 — Protein alignments, corresponding Gblocks html file and CDS sequences for the lineage conserved gametophyte-biased genes. (ZIP 2109 kb) [file 13059_2019_1630_MOESM3_ESM.zip › Con_GA_alignments/Ec-18_001270.aa_ali.fasta-gb.htm]

Ec-18\_001270.aa.fasta.trans.aa\_ali.fasta


## Gblocks 0.91b Results

Processed file: **Ec-18\_001270.aa.fasta.trans.aa\_ali.fasta**  
Number of sequences: **2**  
Alignment assumed to be: **Protein**  
New number of positions: **611** (selected positions are underlined in blue)

```
                         10        20        30        40        50        60
                 =========+=========+=========+=========+=========+=========+
Ec-18_001270     MDPDVADVDGLSAEAAEDISSETRQPEAVALATGYLRQVHDGHGVGSAVDPEESSTGQRK
MSTRG.40855.1    ------------------------------------------------------------
                                                                             


                         70        80        90       100       110       120
                 =========+=========+=========+=========+=========+=========+
Ec-18_001270     PDKTIAGEGTARSAAETADDDVVAEKLPPDDNAPEGGYHVQAGETPAVDATGASTSPDAA
MSTRG.40855.1    ------------------------------------------------------------
                                                                             


                        130       140       150       160       170       180
                 =========+=========+=========+=========+=========+=========+
Ec-18_001270     QLPTLNSGAASLQDSPAPASTTSVSSQGGGGETDEEERSSTEGNQHGQAEPHSSPDEVDN
MSTRG.40855.1    ------------------------------------------------------------
                                                                             


                        190       200       210       220       230       240
                 =========+=========+=========+=========+=========+=========+
Ec-18_001270     SNDLPSKLHRLWGTSPPDRAEGLAVLRNMLGYRKDLDGILSRHVPTKGPRYTRPFHGNDY
MSTRG.40855.1    ------------------------------------------------------------
                                                                             


                        250       260       270       280       290       300
                 =========+=========+=========+=========+=========+=========+
Ec-18_001270     RASSPFSNPRPFTVATLAGRFAPTVTNPSELRERVGTRVRVVTAAGLRVGLARRVFAREP
MSTRG.40855.1    ------------------------------------------------------------
                                                                             


                        310       320       330       340       350       360
                 =========+=========+=========+=========+=========+=========+
Ec-18_001270     LLPSDVEAEEERQSRQQPDASPAPVDVEDLRALVVPVRASASSAPEMSPEGGPAPYPSMA
MSTRG.40855.1    ------------------------------------------------------------
                                                                             


                        370       380       390       400       410       420
                 =========+=========+=========+=========+=========+=========+
Ec-18_001270     EVSSVMWCEYDEDDEEGGGDGGRRRTSAESSPGKKRLLRVSVQTLDGDVCDDEDEDSESD
MSTRG.40855.1    ------------------------------------------------------------
                                                                             


                        430       440       450       460       470       480
                 =========+=========+=========+=========+=========+=========+
Ec-18_001270     DGDSETDVETDIETDFGETEEGSFFDTASPQQGRESTGARGTAAGRSRGGDLSEGGRGNA
MSTRG.40855.1    ------------------------------------------------------------
                                                                             


                        490       500       510       520       530       540
                 =========+=========+=========+=========+=========+=========+
Ec-18_001270     EGARRTSGMRNSKAREEELDEVEYQRKEEVASVLRRLRVAIRRMDGTWTEEKVRSMAKRV
MSTRG.40855.1    ------------------------------------------------------------
                                                                             


                        550       560       570       580       590       600
                 =========+=========+=========+=========+=========+=========+
Ec-18_001270     VRMAVMTASKSELVTTTAARGALRGLGDRTRLRRQQAQRGGAGSGDVGWVTVYVGVGRWG
MSTRG.40855.1    ------------------------------------------------------------
                                                                             


                        610       620       630       640       650       660
                 =========+=========+=========+=========+=========+=========+
Ec-18_001270     PNFFGPGFAVETQEKYRLGWLSLSSQPAAGMMGGGGASGNAPASEQRRVGGAGVRRGAWA
MSTRG.40855.1    ------------------------------------------------------------
                                                                             


                        670       680       690       700       710       720
                 =========+=========+=========+=========+=========+=========+
Ec-18_001270     GRRGFFGLGRRRNSQRREWQDDDEAGIEGWVGSLTCNYCEGHPLGDVPSAGACGEQQGIA
MSTRG.40855.1    ------------------------------------------------------------
                                                                             


                        730       740       750       760       770       780
                 =========+=========+=========+=========+=========+=========+
Ec-18_001270     FKTADSGIRNSPVEQHATSTAVPLNISGEEEGDEVFSEARGTPVTVAESDNDDDRTSQEN
MSTRG.40855.1    ------------------------------------------------------------
                                                                             


                        790       800       810       820       830       840
                 =========+=========+=========+=========+=========+=========+
Ec-18_001270     AAALGSMRTPPNSTLPREASGGSLRVGSGNAVEEKEEQEEWCTGHVCLRLRPDGLRERRR
MSTRG.40855.1    ------------------------------------------------------------
                                                                             


                        850       860       870       880       890       900
                 =========+=========+=========+=========+=========+=========+
Ec-18_001270     GSTSGAGMGLGGDAEEGSEDESGSDTDPASGSDGENLEDLDGIDVEGVSGSTMREPPSEG
MSTRG.40855.1    ------------------------------------------------------------
                                                                             


                        910       920       930       940       950       960
                 =========+=========+=========+=========+=========+=========+
Ec-18_001270     NGRTNRGSRGVPLSSITTSLFEGERRNSGDPCGVSSEKSSKSLEVFAWGDNAGGCLGLPA
MSTRG.40855.1    ------------------------------------------------------------
                                                                             


                        970       980       990      1000      1010      1020
                 =========+=========+=========+=========+=========+=========+
Ec-18_001270     DTSAGLLPRVLQPFGLLPGERVVAVCCSERHSLLVTGMGSVYSAGDGTDGALGLGGRESS
MSTRG.40855.1    ------------------------------------------------------------
                                                                             


                       1030      1040      1050      1060      1070      1080
                 =========+=========+=========+=========+=========+=========+
Ec-18_001270     DEFRLVEWFAEQMPPPKVCQASAGSDLIGCHSAALDADGRLFTWGVGAATGHASLKPVLL
MSTRG.40855.1    ------------------------------------------------------------
                                                                             


                       1090      1100      1110      1120      1130      1140
                 =========+=========+=========+=========+=========+=========+
Ec-18_001270     PREMETLGGFGTGSEARDADGRPAGLRSRAKGVSCGGGFTLAVTNGGKVFAWGSWARGRL
MSTRG.40855.1    ------------------------------------------------------------
                                                                             


                       1150      1160      1170      1180      1190      1200
                 =========+=========+=========+=========+=========+=========+
Ec-18_001270     GLGRPPERTIGRKKKVPRFQGTPRKVSGLGRSPAVQVAAGAWHGMALTEDGEVFTWGHNA
MSTRG.40855.1    --------------------------------------------MALTGDGEVYTWGHNA
                                                             ################


                       1210      1220      1230      1240      1250      1260
                 =========+=========+=========+=========+=========+=========+
Ec-18_001270     SGQLGFLVTPSPASQHRQHQHQPATTNTPTAATSTTSLSEMLRASWTPVKLPAFGK-TSE
MSTRG.40855.1    SGQLGFLVLPSAVSEHQQHRHQTEGVQASTAATSTASLSEMLRASWTPVKLPSFGKGLSD
                 ########################################################  ##


                       1270      1280      1290      1300      1310      1320
                 =========+=========+=========+=========+=========+=========+
Ec-18_001270     PREKLQRAPVADEVL---PG-VGREESLLRKIVHIACGPEHSIAIDSGGAAWTWGAEGRA
MSTRG.40855.1    FPVEPLRAPMAGAGMRAGPGYHGSKRGVRQFATHIACGPEHSIAVDSHGTAWTWGAEGRA
                 ###########                      ###########################


                       1330      1340      1350      1360      1370      1380
                 =========+=========+=========+=========+=========+=========+
Ec-18_001270     CLGHGEAGFGAPGGGTAVAAEAKAKAMGLAGEGGGTDNRQTQTFIPLSREREVSRARARR
MSTRG.40855.1    CLGQGEAGFGAPGEGTAVTAEAQARAMGLPGD---EDRRQQRIFIPLSREREVSRARARR
                 ###############################     ########################


                       1390      1400      1410      1420      1430      1440
                 =========+=========+=========+=========+=========+=========+
Ec-18_001270     ALAGGWAVPREIASLASPP-PPPKTTASNTRDTREG------------------------
MSTRG.40855.1    ALIGGWAVPRRIACLAPTASPLTKTAASNTAGSAEGEGGTAGYVNEEASAAIEMGGVGAG
                 ################    ################                        


                       1450      1460      1470      1480      1490      1500
                 =========+=========+=========+=========+=========+=========+
Ec-18_001270     ---------TGRVKVVAAACGFSHTALVTEDGRMYLFGEGAALAGGDLQALEGENGGVLE
MSTRG.40855.1    ENYGAGGDSSGGAKIVAAAAGFRHTVLVTHDGRMYLFGEGSAVAGGDLSALEGKG-----
                           ###########################################       


                       1510      1520      1530      1540      1550      1560
                 =========+=========+=========+=========+=========+=========+
Ec-18_001270     GTGLREAGGLVRRRCSE--GGEDHGGLDAVAALSAVAGPTPVPRETCSSWFPTLAARRVA
MSTRG.40855.1    AAALRDSGGLVRHQRSEEQGKDQKERPDAVEALSAVAGPTPVPKEVCSAWFPTLAGRRVS
                    ##############  #########################################


                       1570      1580      1590      1600      1610      1620
                 =========+=========+=========+=========+=========+=========+
Ec-18_001270     AVACGGQHAFALLAGDRIGYTLGMDLFRAAMGTERHLPGGPASSERGVLRSGEGSVDEDG
MSTRG.40855.1    AVACAGQHVIVVLAGDHIGYTLGMSLFRAAMGTDRHSFIDAGSE-------GDMDDRRDG
                 ###########################################        #########


                       1630      1640      1650      1660      1670      1680
                 =========+=========+=========+=========+=========+=========+
Ec-18_001270     EGAVGWARGGVDCELLVAGSYLYAHRVVLARRSPVLRDMIAQEERPAISGDD--GDDDGL
MSTRG.40855.1    NGIPTRAKGGVDCELLVGGSYLHGHRVVLASRSPVLRDMIAQEERPGEDSYNGGGSDDWA
                 ##############################################        ######


                       1690      1700      1710      1720      1730      1740
                 =========+=========+=========+=========+=========+=========+
Ec-18_001270     TPPLQLLLPDIRFDVARALLEFLYTGELRRS-LDLDSPLPYDLRAAAKSYGVSRLEVLCT
MSTRG.40855.1    RPPLQLLLPDIRFEVARALLEFLYTGELRRSLFDLGSPLPYDLRAAAKSYGAPRLEALCT
                 ###############################  ###########################


                       1750      1760      1770      1780      1790      1800
                 =========+=========+=========+=========+=========+=========+
Ec-18_001270     EAISLGADPG--EGGP----DAGWGEVVPPPSLAGDLGEALGTMEHADVKFVAGGRPIYA
MSTRG.40855.1    EAISLAADPGRHDGGDGDGGEEGWGKAVPPPSLAGDLGGALGTMEHADVKFVAGGRPIYA
                 ##########            ######################################


                       1810      1820      1830      1840      1850      1860
                 =========+=========+=========+=========+=========+=========+
Ec-18_001270     HRAVLACQSEYFAAMFRFRDQMRRGAADTSSG-RDEHGGHSTAMAEIVVPDSYAGFVRLL
MSTRG.40855.1    HRVLLSCESEYFAAMFRFRDQKGQDGIDIPHGTKEHHEGHATAMAEIVVPDSYSGFVRLL
                 ################################    ########################


                       1870      1880      1890      1900      1910      1920
                 =========+=========+=========+=========+=========+=========+
Ec-18_001270     LFLYTGVLPDANPESVLEDLVCADRYRLLGMKSVSQSMVRLAAGSCLQALEAAEMVNAPL
MSTRG.40855.1    LYIYTGVLPDSGPESLLEDMMSADRYRLLGMKRVCQSMLRLSADSCLQVP----------
                 ################################################            


                       1930      1940      1950      1960      1970      1980
                 =========+=========+=========+=========+=========+=========+
Ec-18_001270     LRQAALFHVEHNLYQVSQQKGFPEIISGTSGVAEVLLSRLHATAANTSAVSSAAARQKRL
MSTRG.40855.1    ------------------------------------------------------------
                                                                             


                       1990      2000      2010      2020      2030      2040
                 =========+=========+=========+=========+=========+=========+
Ec-18_001270     YARSREQRVEADGHGKSNGLVEDGGDPSFRADSPFPWGAALVAVACGTIYYKVSNVVAVG
MSTRG.40855.1    ------------------------------------------------------------
                                                                             


                       2050      2060      2070      2080      2090      2100
                 =========+=========+=========+=========+=========+=========+
Ec-18_001270     MFVPVVNGVFLVCLAAFAFHKIRDKGLFEGGREKRIHRRRQLQQGRGRVVRKRGGWRERR
MSTRG.40855.1    ------------------------------------------------------------
                                                                             


                       2110
                 =========+=======
Ec-18_001270     REAAGGSGRGGSTYLFG
MSTRG.40855.1    -----------------
```

```
Parameters used
Minimum Number Of Sequences For A Conserved Position: 2
Minimum Number Of Sequences For A Flanking Position: 2
Maximum Number Of Contiguous Nonconserved Positions: 8
Minimum Length Of A Block: 10
Allowed Gap Positions: None
Use Similarity Matrices: Yes
```

```
Flank positions of the 13 selected block(s)
Flanks: [1185  1256]  [1259  1271]  [1294  1351]  [1357  1396]  [1401  1416]  [1451  1493]  [1504  1517]  [1520  1603]  [1612  1666]  [1675  1711]  [1714  1750]  [1763  1832]  [1837  1908]  

New number of positions in Ec-18_001270.aa.fasta.trans.aa_ali.fasta-gb:  611  (28% of the original 2117 positions)
```
